# Lower hypoxic ventilatory response in smokers compared to non-smokers during abstinence from cigarettes

**DOI:** 10.1186/s12890-016-0323-0

**Published:** 2016-11-24

**Authors:** Wulf Hildebrandt, Roland Sauer, Ulrich Koehler, Peter Bärtsch, Ralf Kinscherf

**Affiliations:** 1 Former Department of Immunochemistry, German Cancer Research Center (DKFZ), Im Neuenheimer Feld 280, 60120 Heidelberg, Germany; 2 Department of Medical Cell Biology, Institute of Anatomy and Cell Biology, University of Marburg, Robert-Koch-Straße 8, D-35032 Marburg, Germany; 3 Department of Neurology, University Hospital Erlangen, Schwabachanlage 6, 91054 Erlangen, Germany; 4 Sleep Disorder Unit, Department of Pneumology, University Hospital Marburg, Baldingerstraße, 35043 Marburg, Germany; 5 Division VII (Sports Medicine), Medical University Clinic, University of Heidelberg, Im Neuenheimer Feld 410, D-69120 Heidelberg, Germany

**Keywords:** Smoking, Nicotine, Ventilation, Carotid body, Chemoreceptor, O_2_-sensing

## Abstract

**Background:**

Carotid body O_2_-chemosensitivity determines the hypoxic ventilatory response (HVR) as part of crucial regulatory reflex within oxygen homeostasis. Nicotine has been suggested to attenuate HVR in neonates of smoking mothers. However, whether smoking affects HVR in adulthood has remained unclear and probably blurred by acute ventilatory stimulation through cigarette smoke. We hypothesized that HVR is substantially reduced in smokers when studied after an overnight abstinence from cigarettes i.e. after nicotine elimination.

**Methods:**

We therefore determined the isocapnic HVR of 23 healthy male smokers (age 33.9 ± 2.0 years, BMI 24.2 ± 0.5 kg m^−2^, mean ± SEM) with a smoking history of >8 years after 12 h of abstinence and compared it to that of 23 healthy male non-smokers matched for age and BMI.

**Results:**

Smokers and non-smokers were comparable with regard to factors known to affect isocapnic HVR such as plasma levels of glucose and thiols as well as intracellular levels of glutathione in blood mononuclear cells. As a new finding, abstinent smokers had a significantly lower isocapnic HVR (0.024 ± 0.002 vs. 0.037 ± 0.003 l min^−1^ %^−1^BMI^−1^, *P* = 0.002) compared to non-smokers. However, upon re-exposure to cigarettes the smokers’ HVR increased immediately to the non-smokers’ level.

**Conclusions:**

This is the first report of a substantial HVR reduction in abstinent adult smokers which appears to be masked by daily smoking routine and may therefore have been previously overlooked. A low HVR may be suggested as a novel link between smoking and aggravated hypoxemia during sleep especially in relevant clinical conditions such as COPD.

**Electronic supplementary material:**

The online version of this article (doi:10.1186/s12890-016-0323-0) contains supplementary material, which is available to authorized users.

## Background

The peripheral arterial chemoreceptors are key O_2_-sensors for O_2_-homeostasis in normoxia and hypoxia during all phases of life [1–4]. Upon hypoxic stimulation, the chemoreceptors trigger a reflexogenic hypoxic ventilatory response (HVR) which along with neurohumoral responses contributes a great portion to resting minute ventilation limiting arterial O_2_-desaturation e.g. during sleep [1, 2, 5, 6]. The isocapnic HVR, as a measure of carotid O_2_-chemosensitivity, is considered to be a hereditary and therefore relatively stable individual feature and at the same time reveals a surprisingly large interindividual variability [7, 8], which determines and predicts intolerance of healthy subjects to severe hypoxia, e.g. at high altitude [9, 10], and fatal respiratory failure in rare cases of genetically abolished HVR [2]. A low HVR may especially become critical with severely hypoxemic clinical conditions like chronic obstructive pulmonary disease (COPD) or sometimes obstructive sleep apnea [11, 12] which, however, involves a long-term potentiation of HVR with a rather complex pathophysiological role [3, 4, 13]. In healthy adults, a limited number of factors beside drugs reportedly lead to acquired modifications of HVR including acute and chronic hypoxic exposure, aging and, potentially, endurance training [3, 14–17].

A critical HVR attenuation has, however, been extensively discussed to result from long-term intrauterine and/or early postnatal nicotine exposure, thereby possibly linking the sudden infant death syndrome to maternal smoking in a dose–response-fashion [4, 18–21]. Among possible mechanisms suggested for such nicotine-induced impairment of chemoreceptor O_2_-sensing were alterations within the β_2_-subunit of the nicotinic acetylcholine receptor because the nicotine effect was abrogated or mimicked in related β_2_-subunit mutants [22, 23]. In humans, evidence for an impaired chemoreceptor O_2_-sensing through nicotine exposure appears to be preliminary and restricted to studies in infants of smoking mothers in the context of sudden infant death: Healthy, ≤3 months old, term or preterm infants exposed to maternal cigarette smoking/nicotine revealed a weakened (poikilocapnic) HVR and awakening response [18, 24, 25].

However, the important question, whether in adulthood long-term smoking may affect HVR, has remained surprisingly understudied. One earlier study by Kawakami et al. [26] in smokers (SM) and their non-smoking (NSM) homozygote twins failed to demonstrate a smoking-related HVR attenuation after a 3-h-abstinence which is insufficient to eliminate nicotine with an in-vivo half-life of 2 h, as already speculated by these authors themselves. In fact, subsequent studies, including one from the same group, have shown an acute HVR-increase through smoking in both, SM and NSM probably mediated through carotid chemoreceptors [27–30] which might have masked a possible HVR attenuation in the elegant study by Kawakami et al. [26] in twins.

The present study intended to clarify whether or not healthy adult SM reveal a substantial reduction of their isocapnic HVR compared to NSM, when abstaining long enough (12 h overnight) from smoking to eliminate nicotine. According to a representative diurnal profile of smokers, plasma nicotine levels accumulate (to between 10 and 30 ng/ml) in the evening and are eliminated to below 10% within 10 h in healthy adults, while cotinine as a major metabolite is eliminated by about 50% [31, 32]. Furthermore, we evaluated the possible acute masking effect of subsequent re-exposure to cigarette smoke. Because smoking may acutely and chronically induce oxidative stress [33, 34], we also assessed the thiol/disulphide redox state in the plasma and in peripheral blood mononuclear cells (PBMCs), which both may massively affect HVR [35, 36]. In addition, we matched SM and NSM for factors known to affect HVR, like age, sex, and BMI and excluded differences in plasma levels of glucose, HbA1c or potassium [15, 16, 37, 38]. We found a significant, large reduction of isocapnic HVR in healthy male adult SM compared to NSM, which was virtually completely masked by acute enhancement through smoking a single cigarette.

## Methods

### Study design and subjects

To compare HVR between smokers and non-smokers in an observational cross-sectional design, a sample size of 23 per group was calculated for detection of a 30% difference in HVR of the higher value with a power of 80% at the significance level of *P* < 0.05. 46 healthy male subjects were recruited consecutively by public announcements and volunteered for the study. They were assigned to the group of SM (*n* = 23) if they had a >8-years-history of smoking >15 non-mentholated cigarettes /day (>0.5 mg nicotine, >6 mg tar) or to the group of NSM (controls, *n* = 23) if they had no smoking history or regular passive exposure to cigarette smoke according to a detailed initial interview. Health assessment before inclusion into the study included medical history, a physical examination, routine venous blood parameters, pulmonary function test, bilateral brachial arterial blood pressure measurement and a 12-lead electrocardiogram at rest. Exclusion criteria were: blood donation or exposure to an altitude above 2000 m within six months prior to the study, enrollment in competitive sports programs (>6 h per week), any medication or antioxidant supplementation including N-acetylcysteine [35] within the last 3 months, abnormal pulmonary function, hyperlipidemia, hyperhomocysteinemia, arterial hypertension (RR systolic >160 mmHg, diastolic >100 mmHg) or hypotension (RR systolic <90 mmHg), any history or symptom of cardiovascular disease or events, any major intestinal, hepatic, renal, neurological or psychiatric disease, any alcohol or drug abuse, insufficient cooperation and missing oral or written consent.

Informed oral and written consent was obtained from all subjects prior to inclusion into the study, which was approved by the Ethical Committee of the University of Heidelberg (L-264/2001) and performed according to the amended Declaration of Helsinki.

Before measurements all SM were instructed and agreed to abstain from cigarettes for 12 h which was confirmed by a phone call at 11:00 p.m. before the test day and - after an 8-h-sleep - at 7:00 a.m. on test day, before subjects visited the laboratory for blood sampling and HVR measurements between 8:00 and 10:00 a.m. Furthermore, SM as well as NSM avoided any caffeine or drug intake as well as intense physical exercise for 12 h prior to measurements. HVR was determined in duplicate after resuming a comfortable semireclined position for 20 min in a quiet room with ambient temperature between 21 and 23 °C.

### Measurements and equipment

#### Pulmonary function, resting ventilation and HVR

Pulmonary function was assessed by clinical routine parameter (see Table 1) by use of the ‚Flow-Screen‘device (Jaeger, Würzburg, Germany). Resting ventilation (V_E_), inspiratory and end-tidal partial pressure of CO_2_ (PetCO_2_) and O_2_ (PetO_2_) as well as CO_2_-output (VCO_2_) and O_2_-uptake (VO_2_) were measured breath-by-breath by the respiratory monitoring system Oxyconbeta (Mijnhardt, Bunnik, The Netherlands) using the software version 3.12 with elimination of sliding averages. Subjects wore a nose clip and breathed through mouth piece with a flowmeter (Triple V) connected to a low-resistance T-shape valve system (Haward, Edenridge, U.K.) with a dead space of 95 ml. Oxygen saturation (SaO_2_) was measured continuously by a pulse oximeter (3740 Biox Pulse Oximeter, Ohmeda Biox, Louisville, USA) using the finger probe. After recording stable normoxic baseline conditions over 5 min the isocapnic HVR was determined as described [35]. Briefly, HVR was calculated as the slope of the ventilatory response (∆ VE/∆ SaO_2_, ml/min/%) to a progressive lowering SaO_2_ from 100 to 80% (within 6–10 min) which was achieved by progressive admixture of N_2_ to an inspiratory air reservoir with an initial O_2_ fraction of 35%. Thereby PetCO_2_ was kept at individual normoxic baseline levels via CO_2_ admixture by an experienced experimenter.

**Table 1:** Anthropometric data, pulmonary function, normoxic resting ventilation and hypoxic ventilatory response (HVR) in non-smokers (NSM) and smokers (SM)

		NSM	SM^a^	*P*
*n*		23	23	
Cigarettes per day	(n d^−1^)	-	29.6 ± 1.6	-
Pack years^b^	(years)	-	16.8 ± 2.7	-
Age	(years)	32.7 ± 1.7	33.9 ± 2.0	0.644
Body weight	(kg)	78.8 ± 2.5	80.3 ± 1.9	0.632
Body height	(m)	1.80 ± 1.7	1.81 ± 1.2	0.655
BMI	(kg m^−2^)	24.2 ± 0.6	24.2 ± 0.5	0.980
RR systolic	(mmHg)	124.5 ± 1.9	129.6 ± 1.8	0.055
RR diastolic	(mmHg)	79.0 ± 1.9	83.0 ± 1.4	0.93
VC	(l)	5.2 ± 0.2	5.3 ± 0.2	0.847
VC _relative_	(%)	95.9 ± 3.9	95.1 ± 3.0	0.870
FVC _relative_	(%)	108 ± 4	104 ± 4	0.535
FEV1_%VC_	(%)	78.9 ± 2.0	78.0 ± 2.9	0.803
FEV1 _relative_	(%)	106.8 ± 4.8	101.2 ± 4.5	0.407
FEV1_%VC_ _relative_	(%)	97.7 ± 2.7	96.3 ± 3.6	0.744
Ventilation	(l min^−1^)	9.2 ± 0.3	8.0 ± 0.3	0.006^c^
Tidal volume	(ml)	828 ± 56	951 ± 93	0.264
Respirat. frequency	(min^−1^)	12.2 ± 1.0	9.9 ± 0.8	0.078
PetCO_2_	(mmHg)	39.3 ± 0.5	39.2 ± 0.7	0.957
PetO_2_	(mmHg)	101.4 ± 1.1	99.4 ± 1.1	0.218
VCO_2_	(ml min^−1^)	253 ± 8	233 ± 7	0.058
VO_2_	(ml min^−1^)	304 ± 8	291 ± 8	0.268
RQ	(ratio)	0.83 ± 0.01	0.79 ± 0.02	0.088
SaO_2_	(%)	99.1 ± 0.2	99.0 ± 0.2	0.705
HVR	(l min^−1^ %^−1^)	0.89 ± 0.08	0.58 ± 0.05	0.003^c^
HVR ^a^ BMI^−1^	(l min^−1^ %^−1^kg^−1^ m^−2^)	0.037 ± 0.003	0.024 ± 0.002	0.002^c^
PetCO_2 HVR_	(mmHg)	38.8 ± 0.4	38.8 ± 0.6	0.959

Mean ± S.E.M. ^a^>15 cigarettes/day for >8 years with nicotine >0.5 mg and tar >6 mg

^b^cumulative years of 20 cigarettes/day. ^c^ for *P* < 0.01. *BMI* Body mass index, *VC* vital capacity, *FVC* forced vital capacity, *FEV1* forced expiratory volume in 1 s, *PetCO*_*2*_ *and PetCO*_*2*_ end-tidal partial pressures of CO_2_ and O_2_, respectively (BTPS), *VCO*_*2*_ CO_2_ output, *VO*_*2*_ O_2_ uptake, *RQ* Respiratory quotient, i.e. VCO_2_ to VO_2_ ratio, *SaO*_*2*_ peripheral arterial O_2_-saturation, *HVR* hypoxic ventilatory response, *PetCO*_*2 HVR*_ mean PetCO_2_ during HVR measurement

### Venous blood parameters

Postabsorptive blood samples from an anticubital vein were analyzed in the central laboratory of the Medical University Clinic of Heidelberg for plasma levels of triglycerides, total cholesterol, very-low-density-lipoprotein (VLDL), low-density-lipoprotein (LDL), high-density lipoprotein (HDL), glucose (by the hexokinase method of Beckman-Coulter) and HbA1c (by high-performance-liquid-chromatography, HPLC). Commercially available ELISA kits were used to determine levels of oxidized LDL (oxLDL) (Mercodia, Uppsala, Sweden) as well as of tumor-necrosis-factor-α (TNF-α), soluble intercellular- and vascular-adhesion-molecules-1 (sICAM-1 and sVCAM-1) (all provided by IBL, Hamburg, Germany) in EDTA-plasma samples centrifuged at 2000 rpm for 10 min (4 °C) and stored at −75 °C. Total plasma homocysteine was determined immediately by fluorometric detection technique (Abbott Laboratory, Wiesbaden, Germany). The plasma acid-soluble thiol level (mainly cysteine) was measured photometrically (412 nm) as described [35]. The cystine (cysteine-disulphide) concentration was determined from the same supernatant by HPLC technique (Amino Acid Analyzer LC 3000, Eppendorf, Hamburg, Germany). Reduced and total glutathione (GSH) as well as oxidized glutathione (GSSG; glutathione disulphide) were measured in PBMCs isolated by density gradient centrifugation as described [35].

### Statistics

**Fig. 1:**
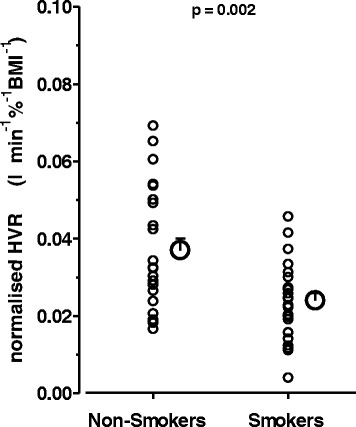
Hypoxic ventilatory response in non-smokers and smokers. Individual (small symbols) and mean ± S.E.M. (big symbols) values of the isocapnic hypoxic ventilatory response (HVR) of non-smokers and 12-h-abstinent smokers. HVR values represent ventilatory increases (l min^−1^) per 1% decrease in O_2_-saturation and are individually normalized for, i.e. divided by, body mass index (BMI). The difference between smokers and non-smokers was highly significant (Student’s t-test, unpaired)

**Fig. 2:**
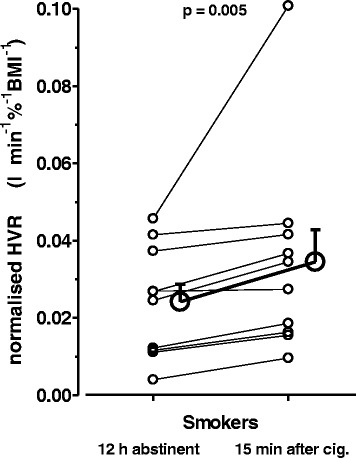
Hypoxic ventilatory response before and after re-exposure to cigarette smoke. Individual (small symbols) and mean ± S.E.M. (big symbols) values of HVR before and after smoking of one cigarette in a subgroup (*n* = 14) of 12-h-abstinent smokers. The increase in HVR through smoking was highly significant (Wilcoxon-test)

All statistical analyses were performed by SPSS (Version 22.0, IBM, Munich, Germany). The main outcome measure HVR as well as secondary variables were compared between SM and NSM by the two-tailed student’s t-test for unpaired samples after testing for normal distribution. Changes in HVR within the group of SM through acute smoking were analyzed by the Wilcoxon-test. All values are presented in figures and tables as means ± SEM, individual values are additionally given in the Figs. 1 and 2. The level of statistical significance was set at *P* < 0.05.

## Results

The anthropometric data of age-matched healthy NSM and SM reflected a normal nutritional status and arterial blood pressure values with no significant differences between the two groups (Table 1). The routine parameters of pulmonary function were comparable between both groups and excluded respiratory diseases of relevance for HVR assessment such as bronchial asthma. Mainly due to a lower, albeit non-significant, respiratory frequency (about −20%, *P* = 0.078), resting minute ventilation was found to be significantly lower in SM (about −13%, *P* = 0.006) compared to NSM. However, this was not associated with any difference in PetCO_2_ levels between the two groups because, at similar VO_2_, SM had almost significantly lower VCO_2_ (about −8%, *P* = 0.058) compared to NSM, i.e., SM tended to have a lower respiratory quotient (RQ, *P* = 0.088). Expectedly, no difference in peripheral arterial O_2_-saturation at rest was detected between SM and NSM.

As a main present finding, isocapnic HVR in terms of both, absolute and normalized values (for individual BMI) showed a highly significant reduction (about −35%) in SM compared to NSM (Fig. 1, Table 1). PetCO_2HVR_ during HVR measurement was well kept at isocapnic levels i.e. at prevailing individual resting normoxic values and, importantly, was virtually identical between SM and NSM (Table 1).

Among the traditional vascular risk factors (Table 2) of these two adult groups, plasma lipids including total cholesterol, VLDL, LDL, HDL, and triglycerides as well as systolic and diastolic arterial blood pressure, fasting glucose, HbA1c and homocysteine were all found to be within the normal range with slightly, though significantly, higher levels observed for triglycerides, total cholesterol, and VLDL in SM compared to NSM. Notably, SM had a considerable and significantly higher level of oxLDL (about 68%) compared to NSM. In contrast, the plasma cysteine and cystine as well as the intracellular GSH and GSSG showed no significant smoking-related differences. While SM showed significantly higher plasma levels of circulating ICAM-1 (about +41%) as another non-traditional cardiovascular risk factor, no significant differences were found for VCAM-1 and TNFα levels compared to NSM.

**Table 2:** Blood cardiovascular risk factors in non-smokers (NSM) and smokers (SM): plasma lipids, oxidized LDL, basal glucose, extra- and intracellular thiol redox state, adhesion molecules and TNF-α

		NSM	SM^a^	*P*
Triglycerides	(mg 100 ml^−1^)	66.4 ± 5.7	94.0 ± 7.5	0.005^c^
Total cholesterol	(mg 100 ml^−1^)	178 ± 7	206 ± 8	0.016^b^
VLDL	(mg 100 ml^−1^)	14.3 ± 1.2	23.1 ± 3.1	0.012^b^
LDL	(mg 100 ml^−1^)	121 ± 7	140 ± 9	0.094
oxLDL	(U l^−1^)	52.9 ± 5.4	88.6 ± 13.6	0.021^b^
HDL	(mg 100 ml^−1^)	44.3 ± 1.8	45.3 ± 2.6	0.734
Glucose	(mg 100 ml^−1^)	79.7 ± 2.8	73.5 ± 2.7	0.115
HbA1c	(%)	5.30 ± 0.07	5.25 ± 0.06	0.590
Homocysteine	(μM)	9.0 ± 0.5	9.2 ± 0.5	0.715
Cysteine	(μM)	7.66 ± 0.32	7.47 ± 0.35	0.687
Cystine	(μM)	40.8 ± 1.1	43.3 ± 1.2	0.126
GSH_intracellular_	(nmol mg^−1^)	15.3 ± 2.2	18.3 ± 1.8	0.303
GSSG_intracellular_	(nmol mg^−1^)	2.94 ± 0.42	2.37 ± 0.50	0.397
ICAM-1	(ng ml^−1^)	378 ± 30	533 ± 35	0.002^c^
VCAM-1	(ng ml^−1^)	791 ± 47	726 ± 40	0.297
TNF-α	(pg ml^−1^)	24.3 ± 4.8	22.4 ± 2.0	0.717

Mean ± S.E.M.; ^a^>15 cigarettes/day for >8 years with nicotine >0.5 mg and tar >6 mg

^b^for *P* < 0.05, ^c^for *P* < 0.01. *OxLDL* oxidized LDL, *GSH* reduced glutathione, *GSSG* glutathione-disulphide in peripheral blood mononuclear cells, *ICAM-1* intercellular adhesion molecule 1, *VCAM-1* vascular adhesion molecule 1, *TNF-α* tumor necrosis factor alpha

In a subgroup of SM (*n* = 10) we furthermore repeated HVR measurement immediately after smoking one cigarette (Fig. 2). This re-exposure led to a highly significant acute increase in HVR (*P* = 0.005) as compared to the condition of 12-h-abstinence from cigarettes. The mean increase in HVR evaluated in SM amounted up to 30% (at a rather wide inter-individual variability), thus reaching a level that was not significantly different from that of NSM (without experimental exposure to cigarette smoke).

According to explorative correlation analysis, the number of ‘pack years’ (range: 6–60) were neither significantly related to the individual HVR (during abstinence from cigarettes or upon re-exposure to one cigarette, with or without normalization for BMI) nor to any other ventilatory parameter given in Table 1. This was also true when controlling for the factor age in a multivariate regression approach. However, a positive correlation of ‘pack years’ was found to SM’s oxLDL (*r* = 0.421, *P* = 0.057) and HbA1_C_ (*r* = 0.475, *P* = 0.022) while a negative correlation existed to intracellular GSSG (*r* = −0.474, *P* = 0.026). The number of daily smoked cigarettes (range: 15–50) showed no significant relation to any measured parameter.

## Discussion

To the best of our knowledge this cross-sectional study is the first to detect a significant and substantial reduction of HVR in healthy, adult long-term SM under conditions of 12-h of abstinence from cigarettes. In addition we demonstrate - well in line with previous findings by others - that upon re-exposure to cigarette smoke HVR is acutely increased to a level that is virtually indistinguishable from that of NSM. This may lead to the important conclusion that a chronic HVR attenuation in SM is obviously masked during daytime smoking habit and therefore may have been overlooked in previous studies with insufficient nicotine abstinence.

The difference in HVR between SM and NSM was demonstrated with a power of 0.97 (*p* ≤ 0.05) at a normal distribution in both groups and a large HVR overlap at the expected wide variability within both the SM and NSM sample (Fig. 1). Unlike the hypercapnic ventilatory response, the isocapnic HVR as a measure of peripheral carotid chemoreceptor O_2_-sensitivity is considered as a quite stable, partly hereditary, individual feature with a uniquely wide inter-subject variability [2, 3, 7, 8] which - in line with a major chemoreceptor contribution to resting ventilator drives [2–5] - is considered to determine (in-) tolerance to high altitude and hypoxemia with pulmonary diseases [1, 6, 9, 10, 12, 13]. Interestingly, smoking may aggravate the physiological O_2_-desaturation during sleep [39]. A low HVR in SM may therefore possibly represent an understudied, novel link between smoking and the risk for aggravated O_2_-desaturation and play a potential role within the complex pathophysiology of COPD or of weaning from artificial respiration. Our finding may therefore warrant more detailed human studies addressing effects of smoking duration, intensity and cessation, possible interaction of gender, aging and additional cardiovascular risk factors, especially those associated with altered HVR like hypertension and obesity [37].

Measurement of ventilator drives in humans is well-known to be easily confounded by several factors, many of which were carefully considered in this study. 1) Isocapnia during HVR was well controlled, i.e. the PetCO_2_ was kept at the level observed during normoxic baseline and was virtually identical between SM and NSM (Table 1). 2) Plasma potassium levels which affect peripheral chemoreceptors were not significantly different between SM and NSM (4.07 ± 0.06 vs 4.32 ± 0.12 mM). 3) We furthermore showed, that the plasma thiol (cysteine and homocysteine) and cystine as well as the intracellular levels of GSH and GSSG were comparable between SM and NSM (Table 2). This is important as interventional studies by us and others have demonstrated a large HVR increase with acute supplementation of thiol-compounds beside a significant correlation between HVR and the intracellular GSH [35, 36]. Though a smoking-related difference in the thiol redox state has previously been reported [33, 34], the present study conducted in a strictly postabsorptive and smoking-abstinent state demonstrated good comparability of the redox state between SM and NSM. Furthermore, SM and NSM had similar whole blood levels of homocysteine, a thiol compound that clearly interacts with other protein- (albumin-) bound thiols like cysteine by disulphide exchange [40]. 4) Another factor influencing HVR is plasma glucose, which was shown to be sensed along with pO_2_ by peripheral chemoreceptor type 1 cells, such, that hypoglycemia massively increases the HVR in humans [38, 41]. The present data were obtained at comparable, fasted blood glucose and HbA1c levels with a tendency towards lower glucose levels in SM, which would rather increase than decreases HVR (Table 2).

Possible mechanisms behind the observed HVR attenuation within the chemoreceptors in adult SM remain speculative at present and may include (epigenetically) altered expression of hypoxia-inducible factor 1α and/or 2α [3], an alteration of the β_2_-nicotinic acetylcholine receptor subunit in the chemoreceptors (or brainstem centers) as a possible target of nicotine [22, 23], dopamine-mediated alterations following an upregulation of the tyrosine hydroxylase within the carotid body as shown in developing rats after nicotine exposure [42], or other factors. Interestingly, HVR in SM at both conditions tested (i.e. during abstinence or upon re-exposure to a single cigarette) was unrelated to pack years (range: 6–60 pack years) or daily smoked cigarettes (range: 15–50), even when controlling for the factor age (range: 22–53 years), which appears to exclude a simple dose-dependent mechanism.

At present, we cannot strictly exclude a rather speculative effect of slight elevations of plasma lipids, oxLDL, or ICAM-1 in SM compared to NSM, because these factors may be associated with endothelial dysfunction, which may not spare out the carotid body arteries. However, HVR was unrelated to these risk factors and considerably higher lipid levels have previously been demonstrated not to affect HVR [43].

As a limitation, this study includes no data on nicotine or cotinine plasma levels to quantify overnight nicotine elimination, i.e. compliance to abstinence from cigarettes or to demonstrate the nicotine increases upon re-exposure to cigarettes. However, our study demonstrates virtually identical plasma thiol (cysteine) levels between SM and NSM on arrival at our laboratory at 8:00 a.m. This may exclude smoking within 1 h prior to blood sampling, because plasma thiol (cysteine) decreases by >50% upon smoking of a single cigarette and takes one hour to return to pre-smoking level [34]. Given that no cigarette was smoked on test day after an 8-h-sleep between 7:00 (reminding phone call) and 8:00 a.m. and that subjects were under observation at the laboratory thereafter until completion of HVR between 9:00 and 10:00 a.m., a 10-h-abstinence from cigarettes can be assumed. The ‘last’ cigarette was reported by phone call or SMS before 11:00 p.m. on the evening before which would yield a 12 h abstinence. In addition, beside the thiol plasma level, our data on the intracellular thiol redox state show similar levels between SM and NSM.

Even with excellent compliance we cannot presently exclude confounding effect of the nicotine metabolites like cotinine (with an in-vivo half-life of around 20 h) and, furthermore, of carboxyhemoglobin (CO-Hb) not detected by the peripheral O_2_-saturation measurement. Because the nicotine clearance depends on various factors including age, gender, hepatic function and blood flow (with large postprandial increase), renal function and factors within the smoking habit itself, further detailed studies on the present observation appear warranted [31, 32]. Thereby, beside the individual smoking history the early childhood cigarette smoke or intrauterine nicotine exposure may have to be assessed as well, to identify relevant factors in smoking-related HVR alterations (chronic reduction as opposed to acute enhancement).

Furthermore, due to a lack of studies in humans, we can only speculate on the finding of an almost significantly lower VCO_2_ and RQ (at similar VO_2_) in SM compared to NSM, which obviously yielded similar PetCO_2_ at significantly lower V_E_ in SM. A previous study in rats has described a (sub-) acute lowering of RQ through nicotine at unchanged resting energy expenditure [44]. Whether this effect is relevant to humans and (still) present (or reversed) upon the presently studied short-term nicotine abstinence, remains unclear at present.

Importantly, the present study at the same time confirmed an acute HVR increase upon (re-) exposure of SM to cigarette smoke to an extent that was sufficient to completely mask the chronic HVR attenuation discussed above (Fig. 2). In fact, the earlier study by Kawakami et al. [26] comparing monozygotic twin SM and NSM, unfortunately, failed to detect differences in HVR, likely because the only 3-h-abstinence from cigarettes used in that study was insufficient to eliminate acute stimulatory affects. Such acute HVR enhancement was, however, subsequently shown, in both SM and NSM as well as for mammals, including one study from the same group [27–30].

## Conclusions

In summary, the present observational study provides evidence for a substantial attenuation of HVR in healthy adult male SM after abstinence from cigarettes, which appears to be masked by (repetitive) smoking during daytime. This may represent an overlooked link between smoking and impaired control of O_2_-homeostasis in SM during times of abstinence like sleep, when behavioral ventilatory drives are minimal [6, 39]. Such impaired protection against O_2_-desaturation in SM may be especially critical in hypoxemic clinical conditions like COPD.

## Additional file

Additional file 1: Supportive information. (XLSX 22 kb)

## Abbreviations

BMI: Body mass index
CO-Hb: Carboxyhemoglobin
COPD: Chronic obstructive pulmonary disease
GSH: Glutathione (reduced)
GSSG: Glutathione disulphide (oxidized)
HDL: High density lipoprotein
HPLC: High performance liquid chromatography
HVR: Hypoxic ventilatory response
LDL: Low-density-lipoprotein
NSM: Non-smokers
oxLDL: Oxidized LDL
PBMC: Peripheral blood mononuclear cells
PetCO_2_: End-tidal partial pressure of CO_2_
PetO_2_: End-tidal partial pressure of O_2_
RQ: Respiratory quotient (VCO_2_ to VO_2_ ratio)
SaO_2_: O_2_-saturation
SEM: Standard error of the mean
sICAM-1: Soluble intercellular adhesion molecule 1
SM: Smokers
sVCAM-1: Soluble vascular adhesion molecule 1
TNFα: Tumor necrosis factor α
VCO_2_: CO_2_ uptake per minute)
V_E_: Ventilation (at rest, per minute)
VLDL: Very-low-density-lipoprotein
VO_2_: O_2_ uptake (per minute)

## References

[1] Blain GM, Smith CA, Henderson KS, Dempsey JA (2009). Contribution of the carotid body chemoreceptors to eupneic ventilation in the intact, unanesthetised dog. J Appl Physiol.

[2] Moore CG, Zwillich CW, Battaglia J, Cottom EK, Weil JV (1976). Respiratory failure associated with familial depression of ventilator responses to hypoxia and hypercarbia. N Engl J Med.

[3] Prabhakar NR (2013). Sensing hypoxia: physiology, genetics and epigenetics. J Physiol.

[4] Prabhakar NR, Peng YJ (2004). Peripheral chemoreceptors in health and disease. J Appl Physiol.

[5] Wade JG, Larson CP, Hickey RF, Ehrenfeld WK, Severinghaus JW (1970). Effect of carotid endarterectomy on the carotid chemoreceptor and baroreceptor function in man. N Engl J Med.

[6] Gries RE, Brooks LJ (1996). Normal oxyhemoglobin saturation during sleep. How low does it go ?. Chest.

[7] Collins DD, Scoggin CH, Zwillich CW, Weil JV (1978). Hereditary aspects of decreased hypoxic response. J Clin Invest.

[8] Weil JV, Bryne-Quinn E, Sodal EI, Friesen WD, Underhill B, Filley GF, Grover RF (1970). Hypoxic ventilatory response in normal men. J Clin Invest.

[9] Richalet JP, Larmignat P, Poitrine E, Letournel M, Canouï-Poitrine F (2002). Physiological risk factors for severe high-altitude illness: a prospective cohort study. Am J Respir Crit Care Med.

[10] Lhuissier FJ, Brumm M, Ramier D, Richalet JP (2012). Ventilatory and cardiac response to hypoxia at submaximal exercise are independent of altitude and exercise intensity. J Appl Physiol.

[11] Kara T, Narkiewicz K, Somers VK (2003). Chemoreflexes - physiology and clinical implications. Acta Physiol Scand.

[12] Osanai S, Akiba Y, Fujiuchi S, Nakano H, Matsumoto H, Ohsaki Y, Kikuchi K (1999). Depression of peripheral chemosensitivity by a dopaminergic mechanism in patients with obstructive sleep apnoea syndrom. Eur Respir J.

[13] Dempsey JA, Veasey SC, Morgan BJ, O’Donell CP (2010). Pathophysiology of sleep apnea. Physiol Rev.

[14] Byrne-Quinn E, Weil JV, Sodal IE, Filley GF, Grover RF (1971). Ventilatory control in the athlete. J Appl Physiol.

[15] Kronenberg RS, Drage CW (1973). Attenuation of the ventilatory and heart rate responses to hypoxia and hypercapnia with aging in normal men. J Clin Invest.

[16] Lhuissier FJ, Canoui-Poitrine F, Richalet JP (2012). Ageing and cardiorespiratory response to hypoxia. J Physiol.

[17] Weil JV, Byrne-Quinn E, Sodal IE, Filley GF, Grover RF (1971). Acquired attenuation of chemoreceptor function in chronically hypoxic man at altitude. J Clin Invest.

[18] Stéphan-Blanchard E, Chardon K, Léké A, Delanaud S, Djeddi D, Libert JP, Bach V, Telliez F (2010). In utero exposure to smoking and peripheral chemoreceptor function in preterm neonates. Pediatrics.

[19] Hafstrom O, Milerad J, Sandberg KL, Sundell HW (2005). Cardiorespiratory effects of nicotine exposure during development. Respir Physiol Neurobiol.

[20] Huang YH, Brown AR, Cross SJ, Cruz J, Rice A, Jaiswal S, Fregosi RF (2010). Influence of prenatal nicotine exposure on development of ventilator response to hypoxia and hypercapnia in neonatal rats. J Appl Physiol.

[21] Nelson EA, Taylor BJ (2001). International child care practices study: infant sleep position and parental smoking. Early Hum Dev.

[22] Cohen G, Roux JC, Grailhe R, Malcolm G, Changeux JP, Lagercrantz H (2005). Perinatal exposure to nicotine causes deficits associated with a loss of nicotinic receptor function. PNAS.

[23] Cohen G, Han ZH, Grailhe R, Gallego J, Gaultier C, Changeux JP, Lagercrantz H (2002). β2 nicotinic acetylcholine receptor subunit modulates protective responses to stress: a --receptor basis for sleep disordered breathing after nicotine exposure. PNAS.

[24] Sovik S, Lossiu K, Walloe L (2001). Heart rate response to transient chemoreceptor stimulation in term infants is modified by exposure to maternal smoking. Pediatr Res.

[25] Ueda Y, Stick SM, Hall G, Sly PD (1999). Control of breathing in infants born to smoking mothers. J Pediatr.

[26] Kawakami Y, Yamamoto H, Yoshikawa T, Shida A (1982). Respiratory chemosensitivity in smokers - studies on monozygotic twin. Am Rev Respir Dis.

[27] Argacha JF, Xhaet O, Gujic M, Adamopoulos D, Beloka S, Dreyfuss C, Degaute JP, van de Borne P (2008). Nicotine increases chemoreflex sensitivity to hypoxia in non-smokers. J Hypertension.

[28] Fernandez R, Larrain C, Zapata P (2002). Acute ventilatory amd circulatory reactions evoked by nicotine: are they excitatory or depressant. Respir Physiol Neurobiol.

[29] Yamamoto H, Inaba S, Nishiura Y, Kishi F, Kawakami Y (1985). Acute inhalation of cigarette smoke augments hypoxic chemosensitivity in humans. J Appl Physiol.

[30] Zapata P, Zuazo A, Llados F (1976). Respiratory and circulatory reflexes induced by nicotine injections: role of carotid body chemoreceptors. Arch Int Pharmacodyn Ther.

[31] Benowitz NL (2010). Nicotine addiction. N Engl J Med.

[32] Molander L, Hansson A, Lunell E (2001). Pharmacokinetics of nicotine in healthy elderly people. Clin Parmacol Ther.

[33] Heitzer T, Brockhoff C, Mayer B, Warnholtz A, Mollnau H, Henne S, Meinertz T, Münzel T (2000). Tetrahydrobiopterin improves endothelium-dependent vasodilation in chronic smokers. Circ Res.

[34] Tsuchiya M, Asada A, Kasahara E, Sato EF, Shindo M, Inoue M (2002). Smoking a single cigarette rapidly reduces combined concentrations of nitrate and nitrite and concentrations of antioxidants in plasma. Circulation.

[35] Hildebrandt W, Alexander S, Bärtsch P, Dröge W (2002). Effect of N-acetyl-cysteine on the hypoxic ventilatory response (HVR) and erythropoietin (EPO) production - linkage between plasma thiol redox state and O_2_ chemosensitivity. Blood.

[36] Lipton AJ, Johnson MA, Macdonald T, Lieberman MW, Gozal D, Gaston B (2001). S-Nitrosothiols signal the ventilatory response to hypoxia. Nature.

[37] Buyse B, Markous N, Cauberghs M, van Klaveren R, Muls E, Demedts M (2003). Effect of obesity and/or sleep apnea on chemosensitivity: differences between men and women. Respir Physiol Neurobiol.

[38] Ward DS, Voter WA, Karan S (2007). The effects of hypo- and hyperglycaemia on the hypoxic ventilatory response in humans. J Physiol.

[39] Casasola GG, Alvarez-Sala JL, Marques JA, Sanchez-Alarcos JM, Tashkin DP, Espinos D (2002). Cigarette smoking behavior and respiratory alterations during sleep in a healthy population. Sleep Breath.

[40] Urquhart BL, House AA, Cutler MJ, Spence JD, Freeman DJ (2006). Thiol exchange: an in vitro assay that predicts the efficacy of novel homocysteine lowering therapies. J Pharm Sci.

[41] Pardal R, López-Barneo J (2002). Low glucose-sensing cells in the carotid body. Nat Neurosci.

[42] Holgert H, Hökfelt T, Hertzberg T, Lagercrantz H (1995). Functional and developmental studies of the peripheral arterial chemoreceptors in rat: effects of nicotine and possible relation to sudden infant death syndrome. Proc Natl Acad Sci U S A.

[43] Barretto-Filho JAS, Consolim-Colombo FM, Guerra-Riccio GM, Santos RD, Chacra AP, Lopes HF, Teixeira SH, Martinez T, Krieger JE, Krieger EM (2003). Hypercholesterolemia blunts forearm vasorelaxation and enhances the pressure response during acute systemic hypoxia. Arterioscler Thromb Vasc Biol.

[44] Bishop C, Parker GC, Coscina DV (2004). Systemic nicotine alters whole-body fat utilization in female rats. Physiol Behav.

